# Supplementary material for: Rydberg electron stabilizes the charge localized state of the diamine cation
Source: Nat Commun. 2024 Jan 4;15:293. doi: 10.1038/s41467-023-44526-y (PMC10767003; doi:10.1038/s41467-023-44526-y)
Supplement: Supplementary file 1 — Supplementary Information [file 41467_2023_44526_MOESM1_ESM.pdf]

Supplementary Information: Rydberg Electron Stabilizes the Charge Localized State of the Diamine Cation

Marc Reimann,[1] Christoph Kirsch,[2] Daniel Sebastiani,[2] and Martin Kaupp*,[1]

[1] *Theoretische Chemie/Quantenchemie, Institut für Chemie, Technische Universität Berlin, 10623 Berlin, Germany.*

[2] *Institut für Chemie, Martin-Luther-University Halle-Wittenberg, 06120 Halle (Saale), Germany*

E-mail: martin.kaupp@tu-berlin.de

Supplementary Discussion 1

To address the performance of the gold standard CCSD(T) of single-reference wave-function methods along the BHandHLYP-generated reaction path (see Methods section in main text for details), we performed single-point calculations at the UHF-UCCSD(T)/aug-cc-pVDZ level of theory, which is very close to the methodology employed in Ref. 1. When inspecting Mulliken spin populations of the resulting UHF wave functions we realized that not one unique, but three qualitatively different UHF-solutions could be found along the path: one in which the positive charge localizes at that nitrogen atom where it also localizes at BHandHLYP level (solution A), one where it is localized at the other nitrogen atom (solution B), and a delocalized solution (solution C) (Figure 1).

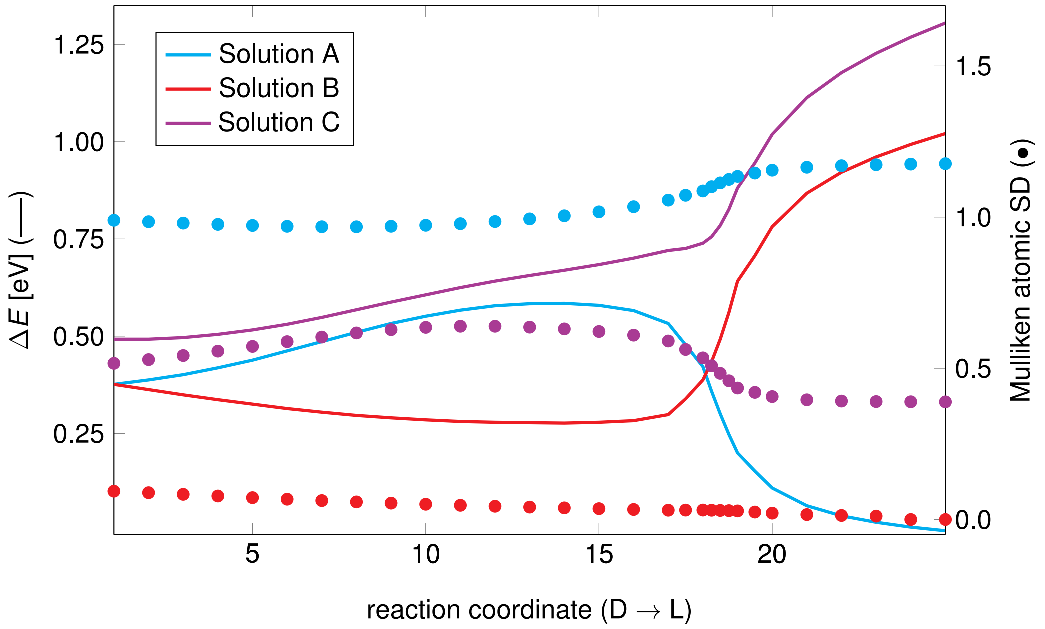

Supplementary Fig. 1: Energies in eV of three different unrestricted Hartree-Fock solutions (UHF/aug-cc-pVDZ) obtained along the pre-generated pathway from the delocalized (D) to the localized (L) structure of dimethylpiperazine (DMP), given relative to the lowest-energy point for solution A (lines, left axis scale). Dots give Mulliken atomic spin densities for the same three solutions at that nitrogen atom, where the positive charge is localized for the original DMP-L^+ calculation (right axis scale). Source data are provided as a Source Data file.

The three UHF solutions show different, unexpected behaviors. The charge-delocalized

solution C, which might be expected to be qualitatively correct at the DMP-D^+ minimum, gives the highest UHF energy at that point. The charge-localized solution A, which gives the qualitatively correct spin densities at the DMP-L^+ minimum, exhibits a very small barrier but then overstabilizes the localized charge compared to the delocalized minimum, leading to a qualitatively wrong shape of the curve. For the left part of the curve, the minimal UHF energy is actually given by the third solution B – contrary to chemical intuition – which leads to a crossing of the solutions A and B. This crossing is in fact the origin of the "barrier" of the overall minimum-energy path at UHF level. Due to the fact that solutions A and B are lower than solution C for the symmetric DMP-D^+ minimum, we identify this as a breaking of spatial symmetry similar to that known for allyl radical[2] (spin-symmetry breaking is minor). Since using ROHF schemes produces almost identical results (see Figs. 1 through 6), the discussion below will only deal with results based on UHF references.

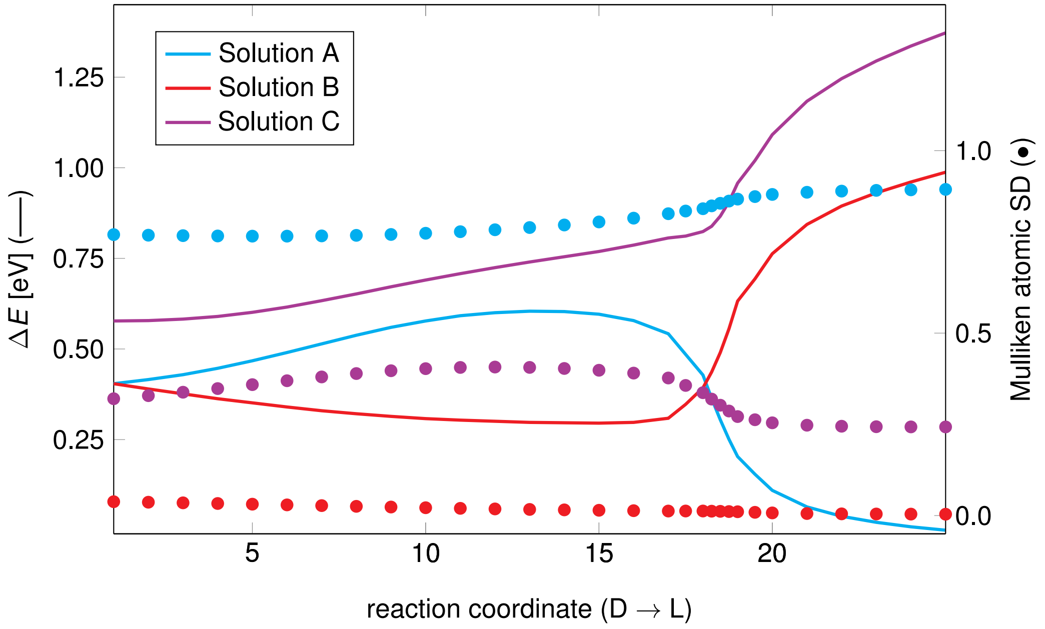

Supplementary Fig. 2: Energies in eV of three different restricted-open shell Hartree-Fock solutions (ROHF/aug-cc-pVDZ) obtained along the pre-generated pathway from the delocalized (D) to the localized (L) structure of dimethylpiperazine (DMP), given relative to the lowest-energy point for solution A (lines, left axis scale). Dots give Mulliken atomic spin densities for the same three solutions at that nitrogen atom, where the positive charge is localized for the original DMP-L^+ calculation (right axis scale). Source data are provided as a Source Data file.

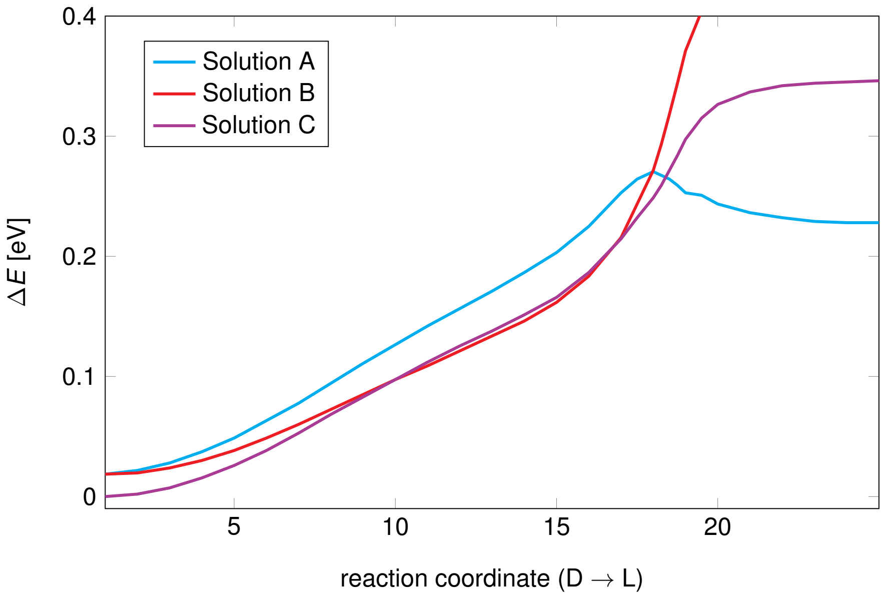

Supplementary Fig. 3: Coupled cluster with single and double excitations energies (CCSD/aug-cc-pVDZ) in eV based on three different unrestricted Hartree-Fock solutions (UHF/aug-cc-pVDZ) obtained along the pre-generated pathway from the delocalized (D) to the localized (L) structure of dimethylpiperazine (DMP), given relative to the lowest-energy point for solution C. Source data are provided as a Source Data file.

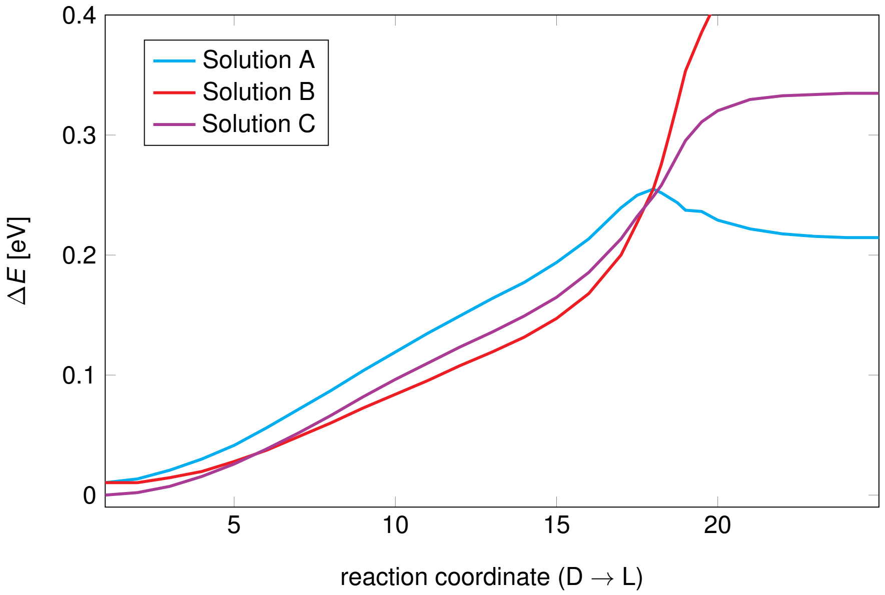

Supplementary Fig. 4: Coupled cluster with single and double excitations energies (CCSD/aug-cc-pVDZ) in eV based on three different restricted-open shell Hartree-Fock solutions (ROHF/aug-cc-pVDZ) obtained along the pre-generated pathway from the delocalized (D) to the localized (L) structure of dimethylpiperazine (DMP), given relative to the lowest-energy point for solution C. Source data are provided as a Source Data file.

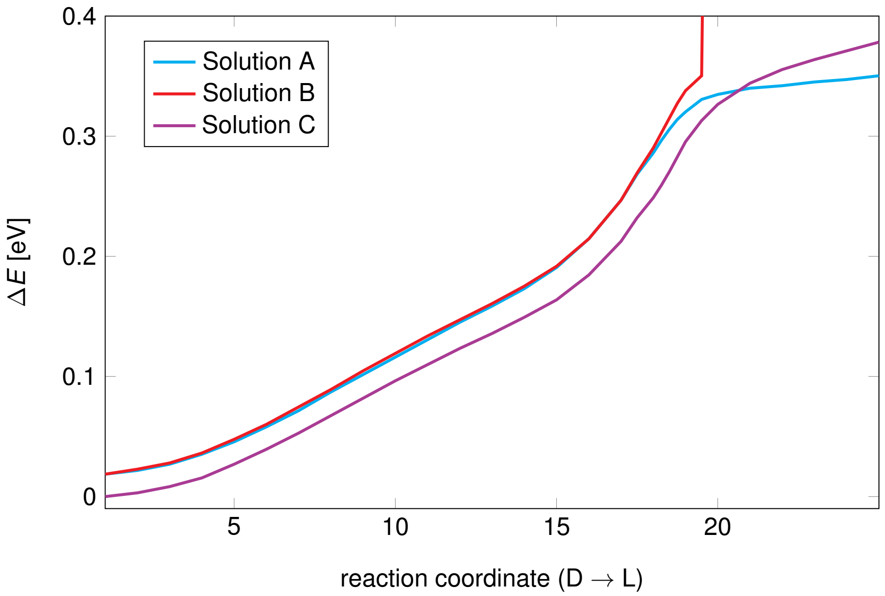

Supplementary Fig. 5: Coupled cluster with single and double and perturbative triple excitations energies (CCSD(T)/aug-cc-pVDZ) in eV based on three different unrestricted Hartree-Fock solutions (UHF/aug-cc-pVDZ) obtained along the pre-generated pathway from the delocalized (D) to the localized (L) structure of dimethylpiperazine (DMP), given relative to the lowest-energy point for solution C. Source data are provided as a Source Data file.

CCSD wavefunctions obtained with the three UHF references compensate this unphysical behavior mostly but not completely. Solution C correctly gives the lowest CCSD energy at the DMP-D^+ structure (Figure 3, left), while the corresponding UHF energy was above the other solutions (see above). We may view this as an example of Löwdin's dilemma of post-Hartee-Fock methods.[3] Solution A gives the correct relative CCSD stabilities of the two end points of the curve and a small artificial maximum (0.04 eV above the "DMP-L^+ region") in exactly the area where UHF solutions A and B cross (cf. Figure 1). The counter-intuitive solution B, which dominated large parts of the UHF surface, is now only marginally lowest in the intermediate region and correctly goes to very high energies in the "DMP-L^+ region". The curves based on UHF solutions B and C change only marginally when perturbative triple substitutions are included at CCSD(T) level (Figure 5). The small local maximum that curve A exhibited at CCSD level is eliminated by the triple-excitation contributions, consistent with earlier work.[4–6]

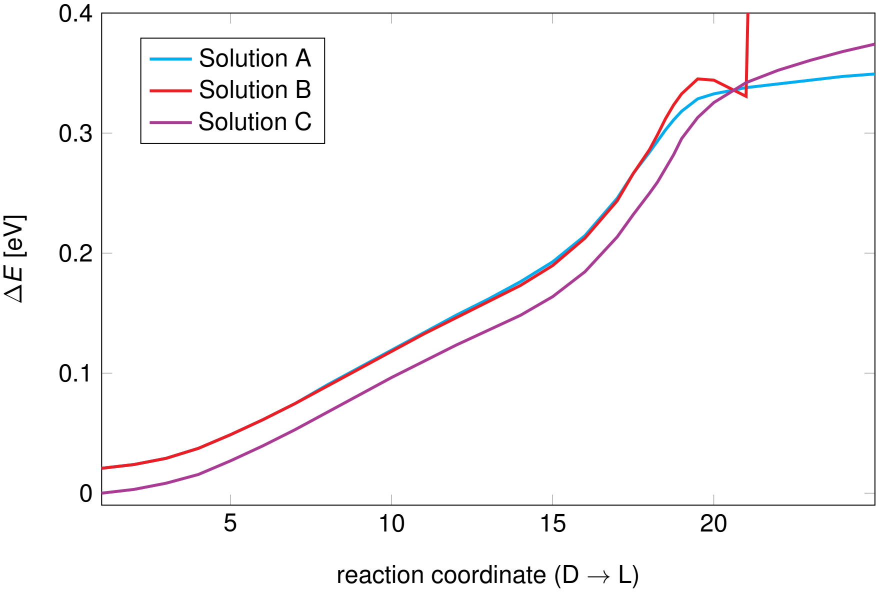

Supplementary Fig. 6: Coupled cluster with single and double and perturbative triple excitations energies (CCSD(T)/aug-cc-pVDZ) in eV based on three different restricted-open shell Hartree-Fock solutions (ROHF/aug-cc-pVDZ) obtained along the pre-generated pathway from the delocalized (D) to the localized (L) structure of dimethylpiperazine (DMP), given relative to the lowest-energy point for solution C. The small negative kink in the curve for solution B indicates two qualitatively different CCSD solutions of significantly different energies. Source data are provided as a Source Data file.

While this has been claimed by some authors[5,6] to be a failure of the perturbative triple-excitation contributions, we do not find this to be the case. Those authors argued by looking at the D_1 diagnostics of the CCSD wave function. We show this quantity along our curves in Figure 7. As can be expected, the D_1 diagnostic changes with the qualitative "correctness" of the underlying UHF solution as it measures the orbital relaxation in the CCSD wave function. It is a measure for the possible importance of triple excitations,[7] but not for a failure of their perturbative treatment. Therefore, a number of authors, e.g. Jiang et al.,[8] have suggested the use of a combined set of different types of diagnostics, including also the leading coefficients of a CASSCF or CISD wave function (C_0) and the contributions of the (T) corrections to the total atomization energies ($\%TAE_e[(T)]$). Using our CASSCF and RASSCF results (for details see below and main text), we find C_0 above 0.96 at CASSCF(11,12) level and above 0.94 at RASSCF(19,4,4;9,1,10) level. The $\%TAE_e[(T)]$ value is in the range 1.3–

1.5 % along the entire path. These indicators are well beyond the multi-reference side of commonly considered thresholds of 0.90 and 10 %,[8] respectively. We are therefore confident that the DMP^+ cation does not exhibit substantial multi-reference character in the relevant parts of the potential-energy surface. If at all, one would expect the multi-reference character to be largest at the "transition state", but then appropriate methods should lower rather than enhance the "barrier".

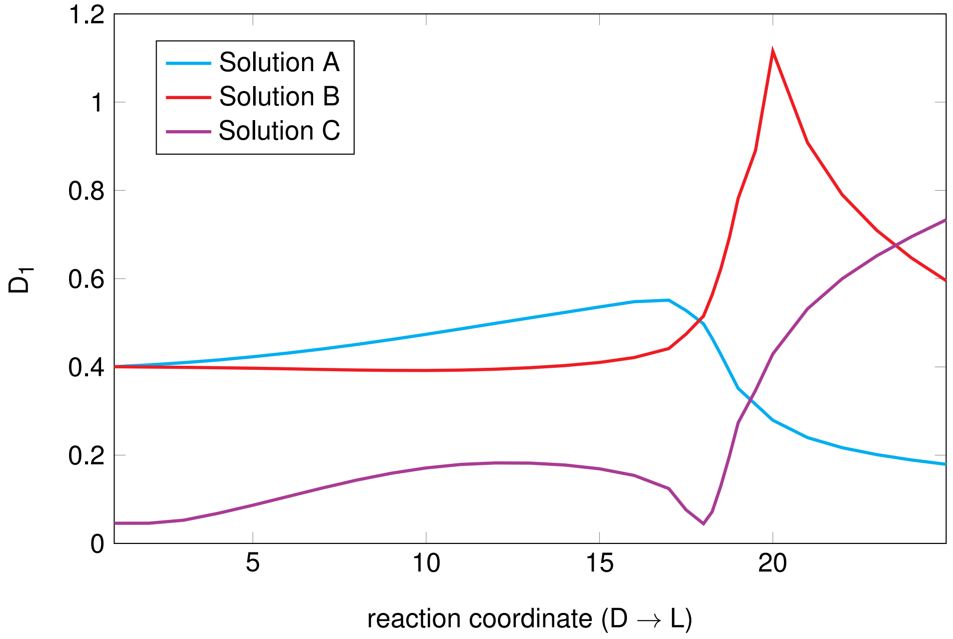

Supplementary Fig. 7: D_1 diagnostics of the coupled cluster with single and double excitations wave functions (CCSD/aug-cc-pVDZ) based on three different unrestricted Hartree-Fock solutions (UHF/aug-cc-pVDZ) obtained along the pre-generated pathway from the delocalized (D) to the localized (L) structure of dimethylpiperazine (DMP). See Figs. 3, 5 for the corresponding CCSD and CCSD(T) energies, respectively. Source data are provided as a Source Data file.

The above discussion indicates that the coupled-cluster energy curves reflect to some extent artefacts arising from the presence of multiple UHF solutions with widely varying behavior along the potential "reaction path". Different remedies are available in such cases. When using a generalized Kohn-Sham (GKS) instead of a UHF reference wave function, symmetry breaking can be reduced by the implicit inclusion of electron correlation in the reference orbitals. This reduces single-excitation contributions, leading to lower D_1 values.

GKS-CCSD and GKS-CCSD(T) results based on BHandHLYP orbitals are shown in Figure 8. As was true for UHF solution A (see above), the CCSD surface shows a small barrier that vanishes upon inclusion of the (T) correction. Unlike the UHF solution, however, the maximum D_1 value along the surface is now 0.08, significantly below those obtained for the HF solution and below critical thresholds. Use of Brueckner orbitals,[9–11] where the D_1 diagnostic vanishes by construction, gives almost identical energy curves. The bias from a specific open-shell reference can be circumvented altogether by employing the IP-EOM-CCSD procedure[12] based on a closed-shell reference. This approach has been used before to circumvent symmetry breaking of the reference wave function.[13] As the IP-EOM-CCSD energy curve is almost identical to the one obtained at BCCD(T) and GKS-CCSD(T) levels (Figure 8), we strongly suspect that the small barriers seen at CCSD levels indeed arise from artificial spatial symmetry breaking of the open-shell reference wave function, while the (T) corrections are physically justified, and the CCSD(T) calculations may be considered to exhibit the correct behavior.

In the context of UHF artefacts generating an artificial cusp, we also scanned the "reaction path" with functionals where the EXX admixture of BHandHLYP-type functionals is varied systematically between 0 % and 100 % in steps of 10 %. The energy curves are compared in Figure 9. A "barrier" is generated when reaching an EXX admixture of 50 % – just the amount present in BHandHLYP – suggesting that the functional inherits the unphysical symmetry breaking of the UHF solutions visible as cusps for larger EXX admixtures. Possibly the semi-local terms in more highly parameterized functionals like M06-HF can to some extent counteract the large UHF contributions and remove the artificial barrier.

Interestingly, we also find no barrier when we use the three different UHF states as a basis for a non-orthogonal configuration interaction (NOCI) treatment,[14] which corrects the unphysical state crossing in the HF solutions.[15] At this level, the qualitatively wrong preference of UHF for DMP-L^+ is also cured (Figure 10). Such a treatment has been shown before to be applicable to related electron-transfer problems.[16]

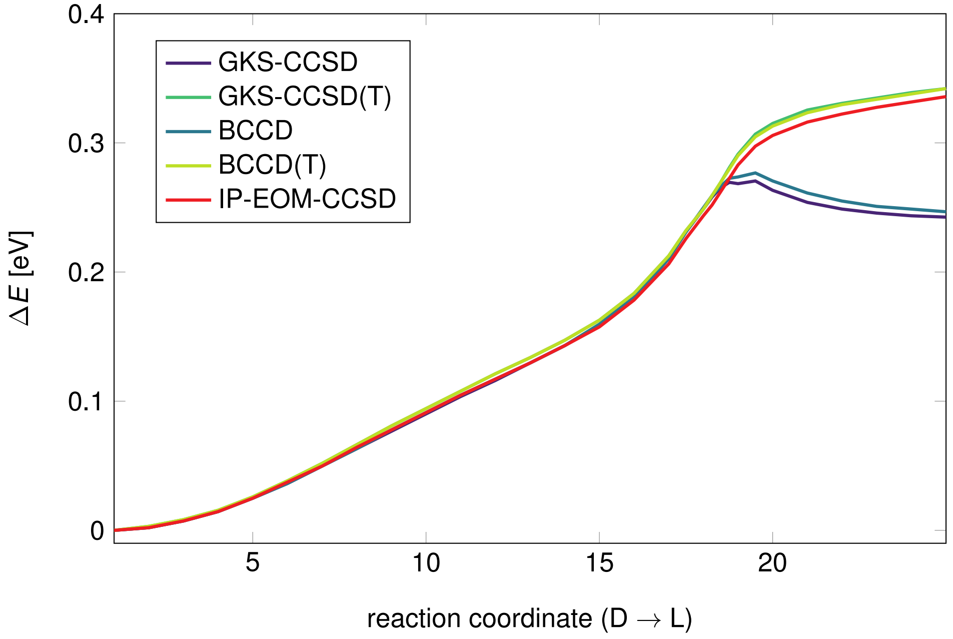

Supplementary Fig. 8: Energy curves in eV (relative to the lowest energy at a given level) obtained along the pre-generated pathway from the delocalized (D) to the localized (L) structure of dimethylpiperazine (DMP) using coupled cluster with single and double excitations (CCSD) and CCSD with perturbative triple excitations (CCSD(T)) using either generalized Kohn–Sham (GKS) or Brückner(B) orbitals or based on the ionization-potential-equation-of-motion (IP-EOM) formalism. Source data are provided as a Source Data file.

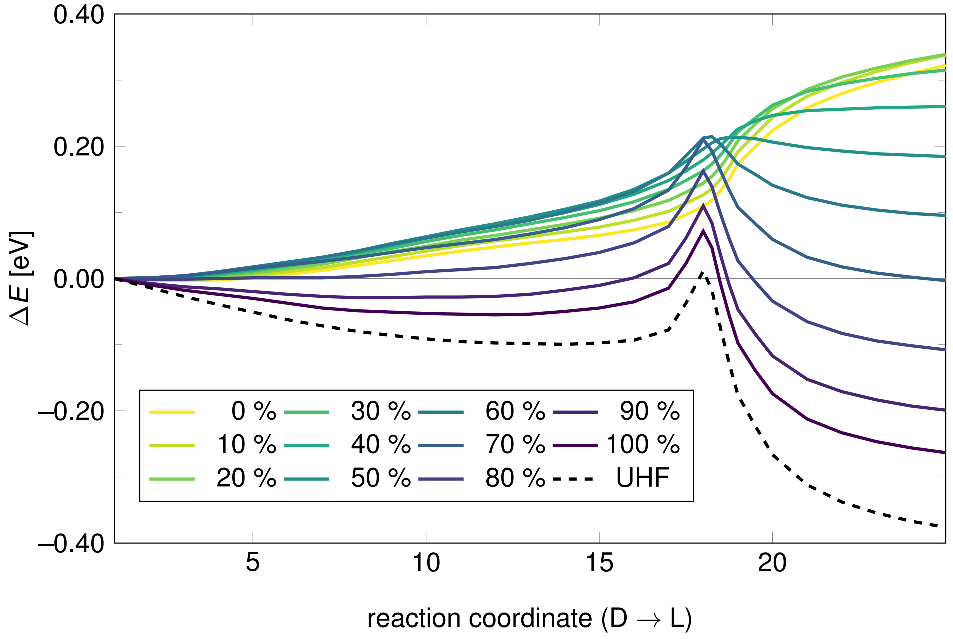

Supplementary Fig. 9: Energy curves in eV along the pre-generated pathway from the delocalized (D) to the localized (L) structure of dimethylpiperazine (DMP) obtained with Becke-1-Lee-Yang-Parr (B1LYP)-type functionals from 0% exact exchange (EXX) admixture (BLYP) to 100% in steps of 10%, each relative to the DMP-D^+ minimum. The corresponding unrestricted Hartree–Fock (UHF) curve is shown for comparison (black dashes). aug-cc-pVDZ results. Source data are provided as a Source Data file.

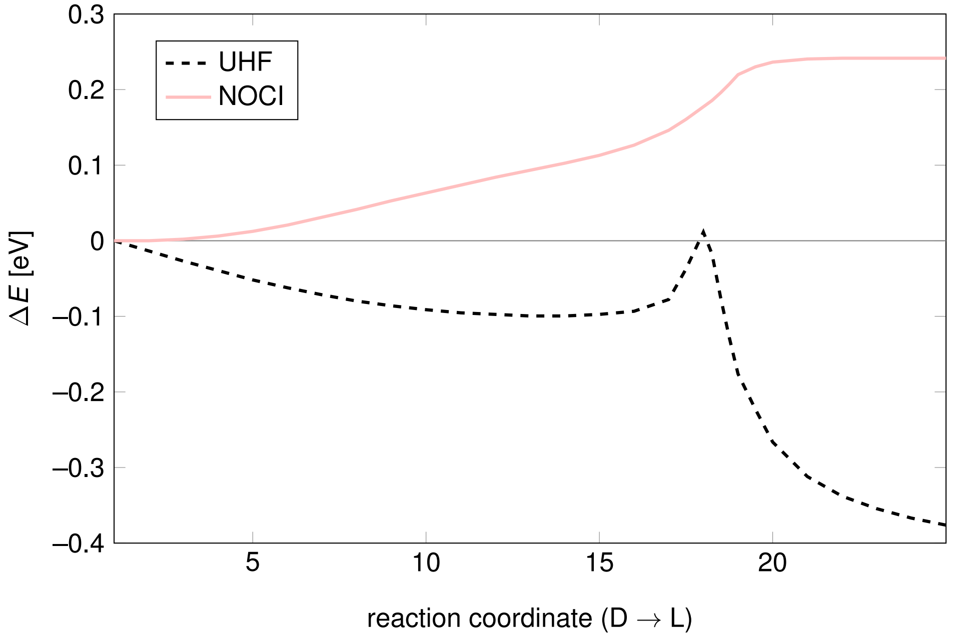

Supplementary Fig. 10: Three-determinant non-orthogonal configuration interaction energy curves (NOCI/aug-cc-pVDZ) in eV compared to the unrestricted Hartree-Fock solution (UHF/aug-cc-pVDZ) obtained along the pre-generated pathway from the delocalized (D) to the localized (L) structure of dimethylpiperazine (DMP), given relative to the DMP-D$^+$ minimum. Source data are provided as a Source Data file.

Supplementary Discussion 2

We have shown above that DMP^+ is not a true multi-reference system. In the main text, we also show problems with true multi-reference methods, namely state-specific CASSCF and MRCI calculations, and how they can me remedied by using state-averaged approaches. All these calculations are based on an CAS(11,12) space, which was modeled for consistency with previous work.[1] An even larger CAS(19,20) space incorporating also the four C-N σ-bonding and four associated σ-antibonding MOs had been suggested in a 2020 preprint.[6] As such a space is too large for a full, conventional CASSCF, the authors used a lower-scaling DMRG[17–19] approach. To study the effects of such large active spaces, we have carried out alternative restricted active space (RAS) computations,[20] where we only include the singly occupied MO in the RAS2 space on top of a larger RAS1 space. When doing this for the smaller 12-orbital space discussed above, this treatment practically converges to identical results as the full CASSCF(11,12) calculations, when up to quadruple excitations from RAS1 and to RAS3 are allowed (see Figure 11). With the same level of substitution allowed for the RAS1 and RAS3 spaces, the extended (19,20) active space results in a smoother energy surface in which the barrier is significantly reduced. Our energy differences between DMP-L^+ and either DMP-D^+ (0.21 eV) or the transition state (0.06 eV) agree reasonably well with the DMRG-CASSCF(19,20) data,[6] 0.24 eV and 0.07 eV, respectively. However, obviously even such a larger active space does not incorporate sufficient dynamical correlation to compete with CCSD(T) calculations. This can be clearly seen in the electronic energies, as the RASSCF energies are only about 0.3 Hartree below the HF energies. Even CCSD gives correlation energies of -1.4 Hartree (which are of course not variationally minimized but should provide the right order of magnitude). An attempt to add such dynamical correlation contributions on top of the DMRG wave function by the NEVPT2 perturbational approach eliminated the barrier completely, and no DMP-L^+ minimum was found.[6] The authors of Ref. 6 blamed the disappearance of the barrier on problems with a perturbational NEVPT2 treatment of dynamical correlation. However, given the very large active space involved,

NEVPT2 should be accurate. The DMRG+NEVPT2 results are fully consistent with the conclusions of the present work regarding the absence of a DMP-L^+ minimum, and with those of Ref. 5 regarding the accuracy of single-reference CCSD(T).

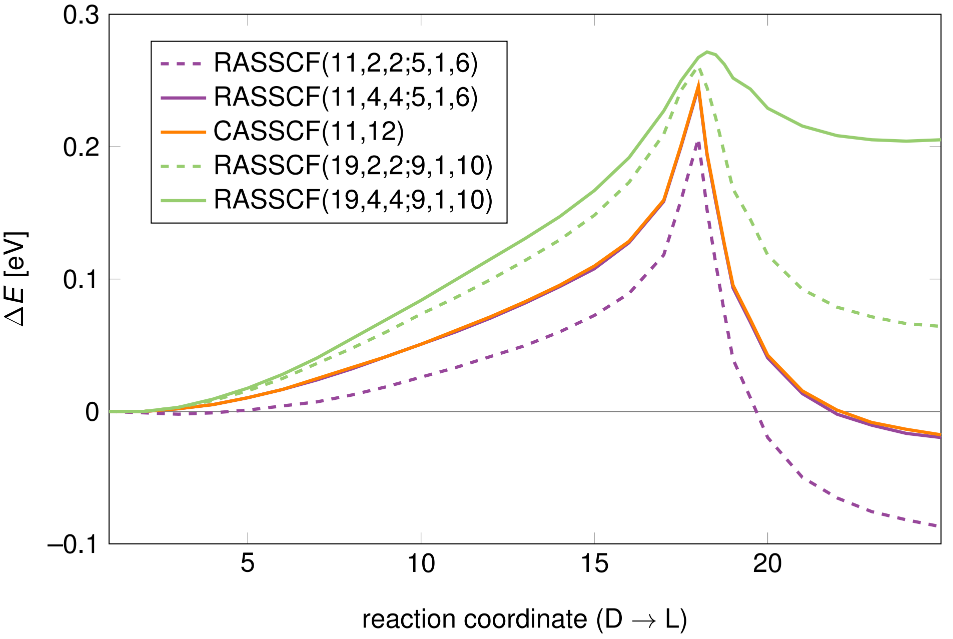

Supplementary Fig. 11: Energies curves in eV (relative to the lowest energy at a given level) along the pre-generated pathway from the delocalized (D) to the localized (L) structure of dimethylpiperazine (DMP) obtained at various restricted-active-space self-consistent field (RASSCF) levels. aug-cc-pVDZ results. Source data are provided as a Source Data file.

To remove the unphysical jump in spin densities observed in state-specific CASSCF calculations, we have also performed state-averaged calculations with the first two doublet states, starting with equal weights. The resulting SA-CASSCF and SA-MRCI+Q curves are depicted in Figure 1 in the main text. These SA-CASSCF and SA-MRCI+Q curves are reproduced in Figure 12 together with the state-specific curves and with curves, where the relative weight of the lower-energy compared to the higher-energy solution at each point is increased to 2:1 and, finally, to 4:1. While the 4:1 CASSCF curve still exhibits remnants of the cusp of the state-specific curve, the 2:1 curve shows already a smoother behavior but still a small barrier. When applying an MRCI+Q treatment on top of these CASSCF calculations (dashed lines), we see that the 4:1 curve still exhibits an exceedingly small maximum and

accordingly a very shallow DMP-L$^+$-type minimum, whereas the 2:1 MRCI+Q curve already increases monotonously from left to right, as does the equally weighted curve. When looking at the nitrogen-atom Mulliken spin density, we see the clearly discontinuous behavior for the state-specific CASSCF being increasingly smoothed out by increased state-mixing. These results provide further support for the artificial (spatial) symmetry-breaking origin of the barrier at the state-specific MRCI+Q level.

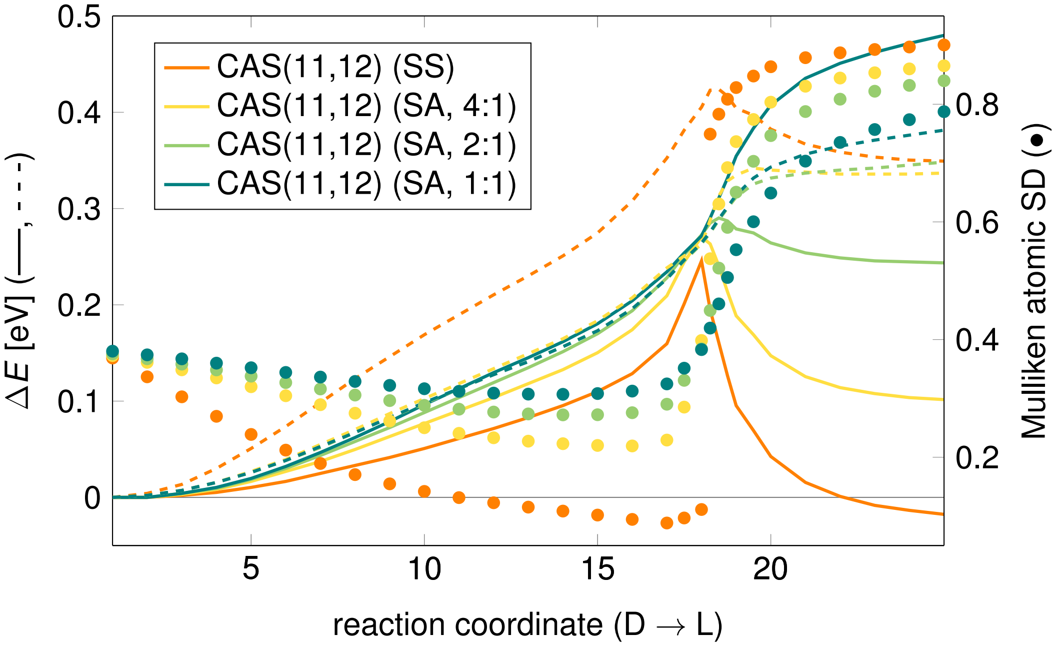

Supplementary Fig. 12: Complete active space (CAS) self-consistent field (CASSCF, solid) and subsequent multi-reference configuration interaction with Davidson correction (MRCI+Q, dashed) energies in eV relative to the delocalized minimum along the pre-generated pathway from the delocalized (D) to the localized (L) structure. Either state-specific (SS, orange) or state-averaged (SA) calculations with different weights of the lower- and higher-energy states (4:1, yellow; 2:1, green; 1:1, teal) are shown. CASSCF Mulliken spin densities at the hole-bearing nitrogen atom in the localized structure are given as dots. A CAS(11,12) space as in Ref. 1 and aug-cc-pVDZ basis sets were used. Source data are provides as a Source data file.

The results shown above used state averaging over the ground state and the first excited state. The second excited state, in which the hole is delocalized over the carbon backbone, is significantly higher in energy (more than 6 eV above the ground state at SA-CASSCF level). Including this state in the state-averaging process consequently affects the results only very

little (see Fig. 13).

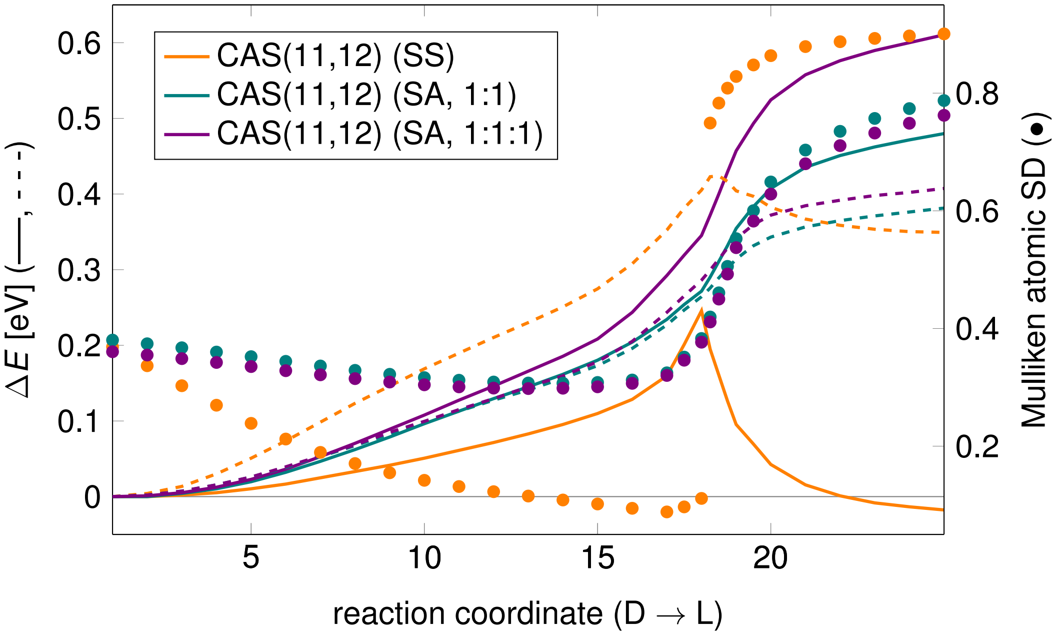

Supplementary Fig. 13: Complete active space (CAS) self-consistent field (CASSCF, solid) and subsequent multi-reference configuration interaction with Davidson correction (MRCI+Q, dashed) energies in eV relative to the delocalized minimum along the pre-generated pathway from the delocalized (D) to the localized (L) structure. Either state-specific (SS, orange) or state-averaged (SA) calculations with equal weights of the ground and first (teal) or first and second (violet) excited states are shown. CASSCF Mulliken spin densities at the hole-bearing nitrogen atom in the localized structure are given as dots. A CAS(11,12) space as in Ref. 1 and aug-cc-pVDZ basis sets were used. Source data are provides as a Source data file.

Supplementary Discussion 3

We analyzed the structural changes of DMP upon ionization to DMP^+ in all obtained AIMD trajectories (see Methods section in main text) at temperatures of 300 K (room temperature) as well as 565 K and 980 K, which are the lowest and highest effective vibrational temperatures after experimental pump excitation and relaxation.[4] DMP^+ rapidly undergoes structural conversion to DMP-D^+ within the first 100 to 200 fs of simulation time, regardless of the choice of the trajectory snapshot at which the ionization was applied. Subsequently, the cation exhibits molecular vibrations around this equilibrium structure but never transforms into a stable DMP-L^+ structure. This behavior is illustrated for one trajectory at each temperature in Figure 14 by means of the two C-N-C-C dihedral angles. The zero in time corresponds to the point at which the ionization DMP $\rightarrow$ DMP^+ was applied. The time evolution of the dihedrals starts from high values of about 170° from the neutral DMP structure, but quickly approaches oscillations around 90°, which is indicative of the DMP-D^+ structure. The combined distribution functions show the frequency of occurrence for each pair of dihedral angle values. The "tails" in this distribution functions in the right of Figure 14 correspond to the relaxation dynamics immediately after ionization. Clearly, the combination of 111° and 168°, which would correspond to DMP-L^+ and is indicated by purple squares, is not observed at 300 K and 565 K and only very rarely at 980 K. With increasing temperature, DMP^+ explores a larger phase space via molecular vibrations, but even at 980 K structures similar to DMP-L^+ do not account for a significant maximum in the combined distribution functions. Moreover, experiments suggest that these localized structures would need to be observed immediately following ionization,[4] which is not the case in our simulations of the cation. Therefore, we conclude that DMP-L^+ is not a minimum on the free energy surface.

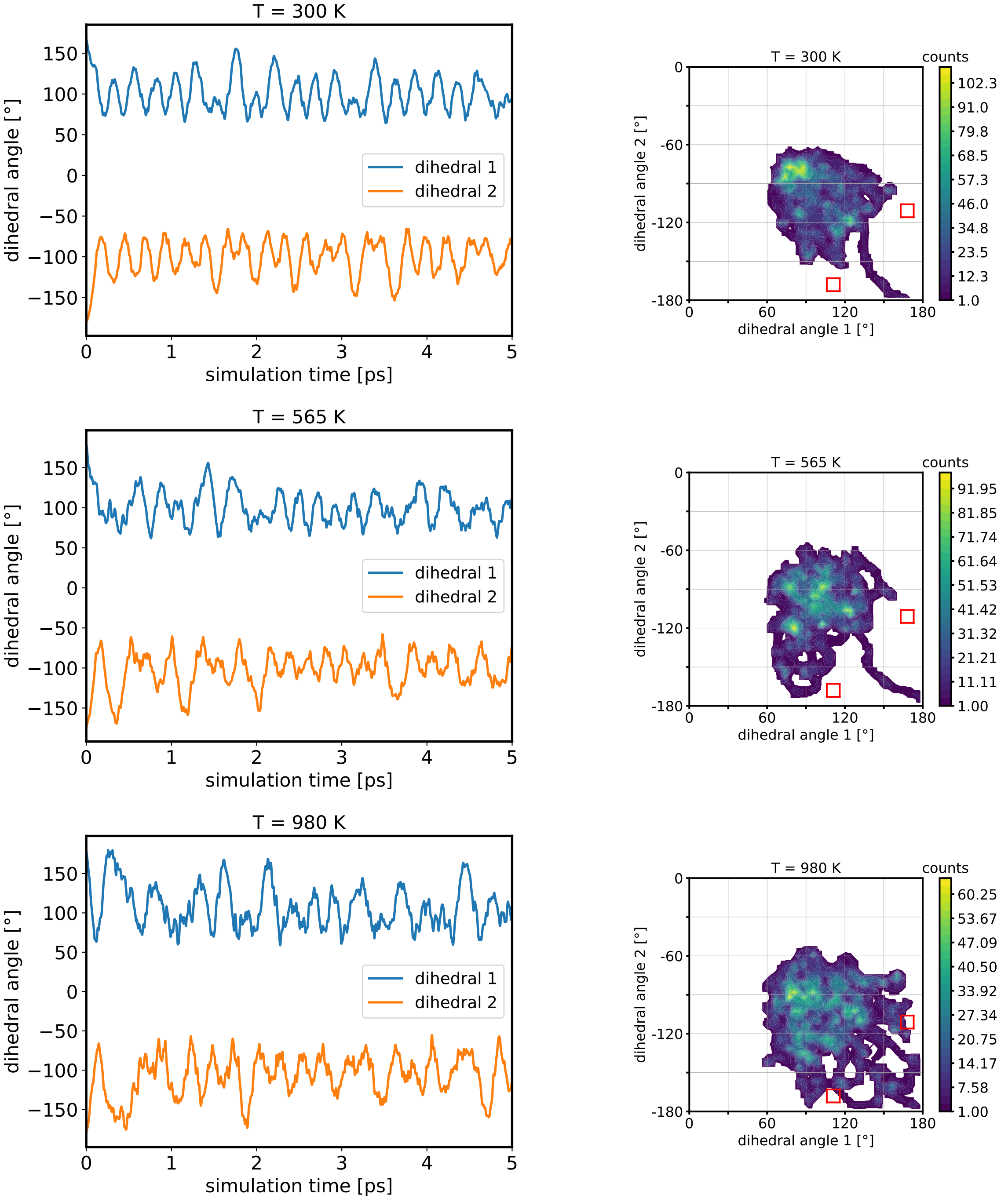

Supplementary Fig. 14: Evolution of the C7-N1-C2-C3 and C8-N4-C5-C6 dihedral angles during simulation time (left) and combined distribution functions[21,22] of their simultaneous occurrence (right) for the dimethylpiperazine (DMP) cation ab-initio molecular dynamics (AIMD) trajectories at T = 300 K, 565 K and 980 K. Dihedrals for the delocalized (DMP-D$^+$) are 2x $\pm 88^\circ$ and for the localized (DMP-L$^+$) $\pm 111^\circ$ and $\pm 168^\circ$ (red squares), respectively (sign arbitrary).

Supplementary Table 1: Electronic energy differences between the delocalized (DMP-D*) and the localized (DMP-L*) minimum and the barrier from the localized side of the Rydberg-state of dimethylpiperazine (DMP) in eV at different levels using def2-TZVPPD basis sets (aug-cc-pVTZ for spin-component scaled coupled cluster (SCS-CC2) calculations). Structures optimized individually at each level. Source data are provided as a Source Data file.

method	$\Delta E_{\mathrm{L}\to\mathrm{D}}$	$\Delta E^{\ddagger}_{\mathrm{L}\to\mathrm{D}}$
TD-PBE-D3(BJ)	−0.372	0.015
TD-PBE0-D3(BJ)	−0.365	0.012
TD-BHandHLYP-D3(BJ)	−0.331	0.028
TD-ωB97X-D	−0.229	0.084
LR-SCS-CC2	−0.236	0.054
**exp.**	−0.22 ± 0.04[a]	≈ 0.1[b]

[a] ΔH obtained from temperature dependent equilibrium constants, taken from Ref. 4. [b] Rough estimate based on the reaction kinetics, taken from Ref. 23.

Supplementary Table 2: Vertical binding energies of the Rydberg electron in the delocalized (DMP-D*) and the localized (DMP-L*) Rydberg-state of dimethylpiperazine (DMP) and their relative differences in eV at different levels using def2-TZVPPD basis sets (aug-cc-pVTZ for coupled cluster methods). Structures optimized individually at each level. Source data are provided as a Source Data file.

method	$\Delta E_{\mathrm{DMP\text{-}D^*}}$	$\Delta E_{\mathrm{DMP\text{-}L^*}}$	$\Delta\Delta E$
TD-PBE-D3(BJ)	3.01	3.17	+0.16
TD-PBE0-D3(BJ)	2.45	2.58	+0.12
TD-BHandHLYP-D3(BJ)	2.01	1.96	−0.05
TD-ωB97X-D	1.98	2.25	+0.27
LR-SCS-CC2	2.64	2.98	+0.34
IP-EOM-CCSD[a]	2.54	2.65	+0.11
**exp.**[4]	2.70 ± 0.03	2.81 ± 0.04	+0.11 ± 0.07

[a] At LR-SCS-CC2 structures.

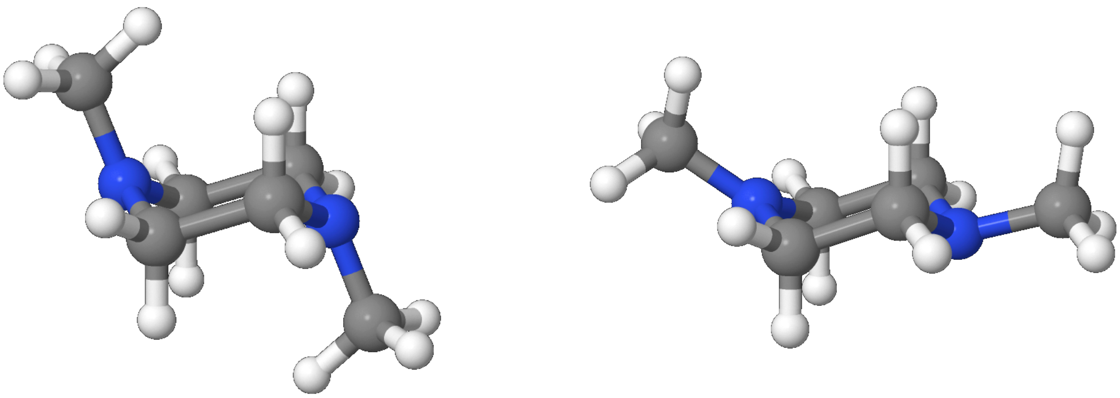

Supplementary Fig. 15: Structures of the two isomers of the Rydberg state of dimethylpiperazine (DMP) (left: delocalized (DMP-D*), right: localized(DMP-L*)). Structures were optimized at the linear-response spin-component scaled coupled cluster level (LR-SCS-CC2/aug-cc-pVTZ).

Supplementary Table 3: Bond lengths in the delocalized (DMP-D*) and localized (DMP-L*) Rydberg states of dimethylpiperazine (in Å) obtained at linear-response spin-component scaled coupled cluster level of theory (LR-SCS-CC2/aug-cc-pVTZ) compared to X-ray scattering data from Ref. 24.

	LR-SCS-CC2	exp.[a]
	DMP-D*	
$d_{\text{N-C(CH}_3\text{)}}$	1.462	1.480
$d_{\text{N-C(CH}_2\text{)}}$	1.427	1.465
$d_{\text{C(CH}_2\text{)-C(CH}_2\text{)}}$	1.600	1.634
$d_{\text{N-N}}$	2.816	2.986
$d_{\text{C(CH}_3\text{)-C(CH}_3\text{)}}$	4.994	5.200
	DMP-L*	
$d_{\text{N-C(CH}_3\text{)}}$	1.462	1.478
$d_{\text{N-C(CH}_2\text{)}}$	1.458	1.505
$d_{\text{C(CH}_2\text{)-C(CH}_2\text{)}}$	1.535	1.571
$d_{\text{N-N}}$	2.804	2.904
$d_{\text{C(CH}_3\text{)-C(CH}_3\text{)}}$	5.473	5.311

[a]These data are based on substantial information from previous calculations, e.g. regarding electron-density changes compared to the DMP ground state. Sub-Å accuracy has been reported, we expect the error margins to be at least comparable to those of the present LR-SCS-CC2 calculations. The larger discrepancies of the distances between opposing methyl carbon atoms likely reflect a large-amplitude relative motion of the methyl groups.

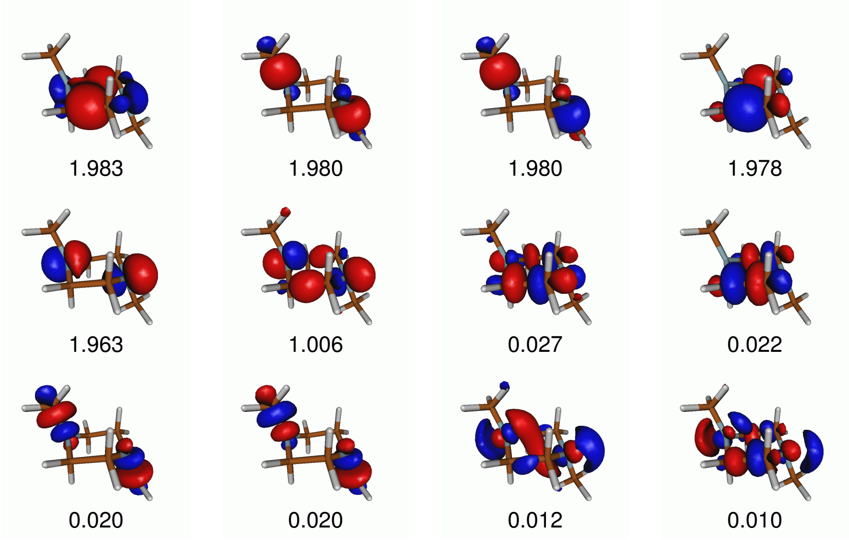

Supplementary Fig. 16: Contour plots (isovalue ±0.05) and occupation numbers of the natural orbitals obtained at the state-specific complete-active-space self-consistent-field (SS-CASSCF(11,12)) level for the delocalized cation of dimethylpiperazine (DMP-D$^+$) with aug-cc-pVDZ basis.

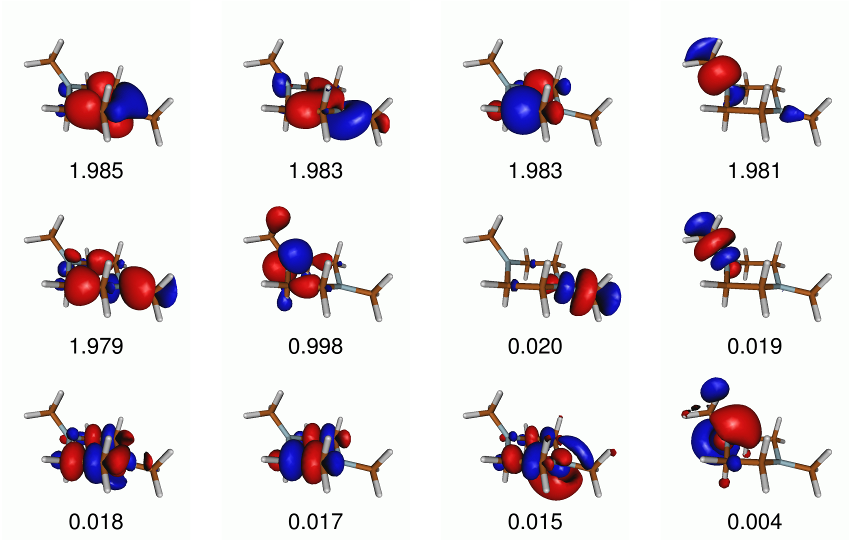

Supplementary Fig. 17: Contour plots (isovalue ±0.05) and occupation numbers of the natural orbitals obtained at the state-specific complete-active-space self-consistent-field (SS-CASSCF(11,12)) level for the localized cation of dimethylpiperazine (DMP-L$^+$) with aug-cc-pVDZ basis.

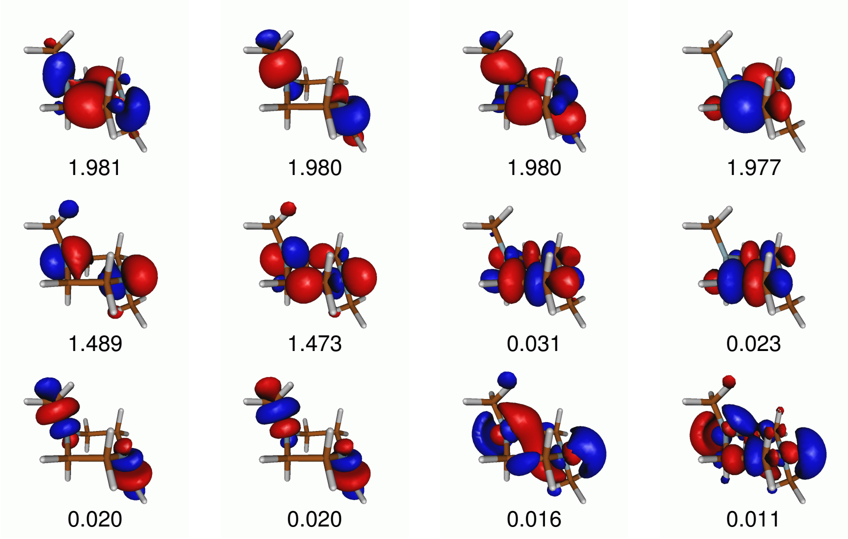

Supplementary Fig. 18: Contour plots (isovalue ±0.05) and occupation numbers of the natural orbitals obtained at the state-averaged complete-active-space self-consistent-field (SA-CASSCF(11,12)) level for the delocalized cation of dimethylpiperazine (DMP-D$^+$) using the first two doublet states and equal weights, with aug-cc-pVDZ basis.

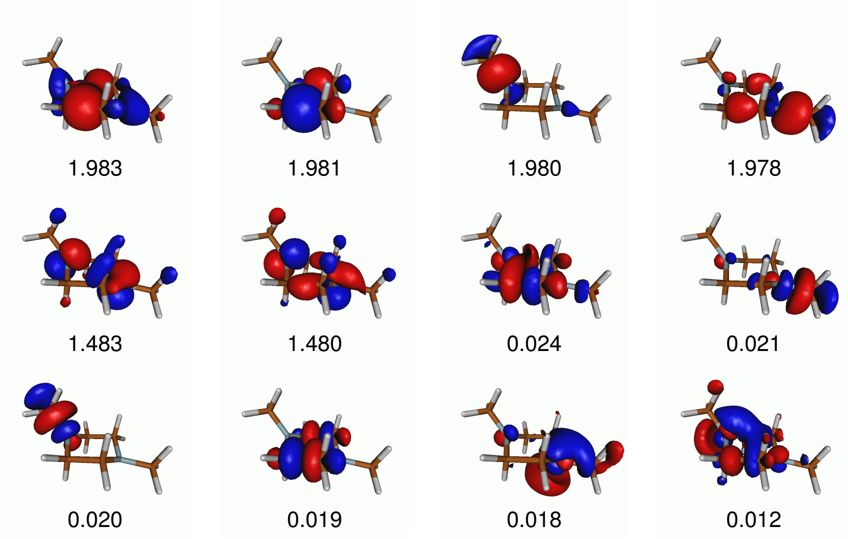

Supplementary Fig. 19: Contour plots (isovalue ±0.05) and occupation numbers of the natural orbitals obtained at the state-averaged complete-active-space self-consistent-field (SA-CASSCF(11,12)) level for the localized cation of dimethylpiperazine (DMP-L$^+$) using the first two doublet states and equal weights, with aug-cc-pVDZ basis.

Supplementary References

(1) Gałyńska, M.; Ásgeirsson, V.; Jónsson, H.; Bjornsson, R. Localized and Delocalized States of a Diamine Cation: Resolution of a Controversy. *J. Phys. Chem. Lett.* **12**, 1250–1255 (2021).

(2) McKelvey, J.; Hehre, W. J. Symmetry Dilemma in All-Electron MO Theory of the Allyl Radical. *Mol. Phys.* **25**, 983–984 (1973).

(3) Lykos, P.; Pratt, G. W. Discussion on The Hartree-Fock Approximation. *Rev. Mod. Phys.* **35**, 496–501 (1963).

(4) Cheng, X.; Zhang, Y.; Jónsson, E.; Jónsson, H.; Weber, P. M. Charge Localization in a Diamine Cation Provides a Test of Energy Functionals and Self-Interaction Correction. *Nat. Commun.* **7**, 11013 (2016).

(5) Ali, Z. A.; Aquino, F. W.; Wong, B. M. The Diamine Cation Is Not a Chemical Example where Density Functional Theory Fails. *Nat. Commun.* **9**, 4733 (2018).

(6) Gałyńska, M.; Ásgeirsson, V.; Jónsson, H.; Bjornsson, R. Localized and delocalized states of a diamine cation: A critical test of wave function methods. Preprint at `https://arxiv.org/abs/2007.06125` (2020).

(7) Leininger, M. L.; Nielsen, I. M. B.; Crawford, T. D.; Janssen, C. L. A New Diagnostic for Open-Shell Coupled-Cluster Theory. *Chem. Phys. Lett.* **328**, 431–436 (2000).

(8) Jiang, W.; DeYonker, N. J.; Wilson, A. K. Multireference Character for 3d Transition-Metal-Containing Molecules. *J. Chem. Theory Comput.* **8**, 460–468 (2012).

(9) Chiles, R. A.; Dykstra, C. E. An Electron Pair Operator Approach to Coupled Cluster Wave Functions. Application to He_2, Be_2, and Mg_2 and Comparison with CEPA Methods. *J. Chem. Phys.* **74**, 4544–4556 (1981).

(10) Handy, N. C.; Pople, J. A.; Head-Gordon, M.; Raghavachari, K.; Trucks, G. W. Size-Consistent Brueckner Theory Limited to Double Substitutions. *Chem. Phys. Lett.* **164**, 185–192 (1989).

(11) Watts, J. D.; Gauss, J.; Bartlett, R. J. Coupled-Cluster Methods with Noniterative Triple Excitations for Restricted Open-Shell Hartree–Fock and Other General Single Determinant Reference Functions. Energies and Analytical Gradients. *J. Chem. Phys.* **98**, 8718–8733 (1993).

(12) Stanton, J. F.; Gauss, J. Analytic Energy Derivatives for Ionized States Described by the Equation-of-Motion Coupled Cluster Method. *J. Chem. Phys.* **101**, 8938–8944 (1994).

(13) Kaldor, U. The Ground State Geometry of the NO_3 Radical. *Chem. Phys. Lett.* **166**, 599–601 (1990).

(14) Malmqvist, P. Å. Calculation of Transition Density Matrices by Nonunitary Orbital Transformations. *Int. J. Quantum Chem.* **30**, 479–494 (1986).

(15) Thom, A. J. W.; Head-Gordon, M. Hartree–Fock solutions as a quasidiabatic basis for nonorthogonal configuration interaction. *J. Chem. Phys.* **131**, 124113 (2009).

(16) Jensen, K. T.; Benson, R. L.; Cardamone, S.; Thom, A. J. W. Modeling Electron Transfers Using Quasidiabatic Hartree–Fock States. *J. Chem. Theory Comput.* **14**, 4629–4639 (2018).

(17) White, S. R.; Martin, R. L. Ab Initio Quantum Chemistry Using the Density Matrix Renormalization Group. *J. Chem. Phys.* **110**, 4127–4130 (1999).

(18) Mitrushenkov, A. O.; Fano, G.; Ortolani, F.; Linguerri, R.; Palmieri, P. Quantum Chemistry Using the Density Matrix Renormalization Group. *J. Chem. Phys.* **115**, 6815–6821 (2001).

(19) Wouters, S.; Van Neck, D. The Density Matrix Renormalization Group for ab Initio Quantum Chemistry. *Eur. Phys. J. D* **68**, 272 (2014).

(20) Malmqvist, P. A.; Rendell, A.; Roos, B. O. The Restricted Active Space Self-Consistent-Field Method, Implemented with a Split Graph Unitary Group Approach. *J. Phys. Chem.* **94**, 5477–5482 (1990).

(21) Brehm, M.; Kirchner, B. TRAVIS - A Free Analyzer and Visualizer for Monte Carlo and Molecular Dynamics Trajectories. *J. Chem. Inf. Model.* **51**, 2007–2023 (2011).

(22) Brehm, M.; Thomas, M.; Gehrke, S.; Kirchner, B. TRAVIS - A Free Analyzer for Trajectories from Molecular Simulation. *J. Chem. Phys.* **152**, 164105 (2020).

(23) Cheng, X.; Jónsson, E.; Jónsson, H.; Weber, P. M. Reply to: "The Diamine Cation is not a Chemical Example where Density Functional Theory Fails". *Nat. Commun.* **9**, 5348 (2018).

(24) Yong, H.; Xu, X.; Ruddock, J. M.; Stankus, B.; Carrascosa, A. M.; Zotev, N.; Bellshaw, D.; Du, W.; Goff, N.; Chang, Y.; Boutet, S.; Carbajo, S.; Koglin, J. E.; Liang, M.; Robinson, J. S.; Kirrander, A.; Minitti, M. P.; Weber, P. M. Ultrafast X-Ray Scattering Offers a Structural View of Excited-State Charge Transfer. *Proc. Natl. Acad. Sci.* **118**, e2021714118 (2021).
