# Supplementary material for: Rydberg electron stabilizes the charge localized state of the diamine cation
Source: Nat Commun. 2024 Jan 4;15:293. doi: 10.1038/s41467-023-44526-y (PMC10767003; doi:10.1038/s41467-023-44526-y)
Supplement: Supplementary file 3 — Description of additional supplementary information [file 41467_2023_44526_MOESM3_ESM.pdf]

Description of Additional Supplementary Files

File Name: Supplementary Data 1
Description:

Optimized atomic coordinates for the reaction pathway from the delocalized to the localized minimum of the dimethylpiperazine cation at the BHandHLYP+D3(BJ)/def2-TZVPP level. All structures are given in the order in which they were used throughout the manuscript. Structures are given in .xyz file format.

File Name: Supplementary Data 2
Description:

Optimized atomic coordinates for the reaction pathway from the delocalized to the localized minimum of the dimethylpiperazine Rydberg state at the LR-SCS-CC2/aug-cc-pVTZ level. All structures are given in the order in which they were used throughout the manuscript. Structures are given in .xyz file format.

File Name: Supplementary Data 3
Description:

Total electronic energies for each state for all performed state-averaged CASSCF calculations. Energies are given in .xslx file format.

File Name: Supplementary Data 4
Description:

Initial and final atomic coordinates for our molecular dynamics simulation runs performed at the PBE0+D3(BJ)/DZVP-MOLOPT-GTH level. Structures are given in .xyz file format.
